# Perceptions of Adolescents With Cancer Related to a Pain Management App and Its Evaluation: Qualitative Study Nested Within a Multicenter Pilot Feasibility Study

**DOI:** 10.2196/mhealth.9319

**Published:** 2018-04-06

**Authors:** Lindsay A Jibb, Bonnie J Stevens, Paul C Nathan, Emily Seto, Joseph A Cafazzo, Donna L Johnston, Vanessa Hum, Jennifer N Stinson

**Affiliations:** ^1^ School of Nursing Faculty of Health Sciences University of Ottawa Ottawa, ON Canada; ^2^ Evidence-to-Practice Program Children's Hospital of Eastern Ontario Research Institute Ottawa, ON Canada; ^3^ Child Health Evaluative Sciences Program Hospital for Sick Children Toronto, ON Canada; ^4^ Lawrence S Bloomberg Faculty of Nursing University of Toronto Toronto, ON Canada; ^5^ Division of Hematology/Oncology Hospital for Sick Children Toronto, ON Canada; ^6^ Institute of Health Policy, Management and Evaluation University of Toronto Toronto, ON Canada; ^7^ eHealth Innovation University Health Network Toronto, ON Canada; ^8^ Division of Hematology/Oncology Children's Hospital of Eastern Ontario Ottawa, ON Canada; ^9^ Faculty of Medicine University of Ottawa Ottawa, ON Canada; ^10^ Think Research Corporation Toronto, ON Canada; ^11^ Department of Anesthesia and Pain Medicine Hospital for Sick Children Toronto, ON Canada

**Keywords:** pain, adolescent, cancer, supportive care, mHealth, qualitative

## Abstract

**Background:**

Pain in adolescents with cancer is common and negatively impacts health-related quality of life. The Pain Squad+ smartphone app, capable of providing adolescents with real-time pain management support, was developed to enhance pain management using a phased approach (ie, systematic review, consensus conference and vetting, iterative usability testing cycles). A 28-day Pain Squad+ pilot was conducted with 40 adolescents with cancer to evaluate the feasibility of implementing the app in a future clinical trial and to obtain estimates of treatment effect.

**Objective:**

The objective of our nested qualitative study was to elucidate the perceptions of adolescents with cancer to determine the acceptability and perceived helpfulness of Pain Squad+, suggestions for app improvement, and satisfaction with the pilot study protocol.

**Methods:**

Post pilot study participation, telephone-based, semistructured, and audio-recorded exit interviews were conducted with 20 adolescents with cancer (12-18 years). All interviews were transcribed and independently coded by 2 study team members. Content analysis was conducted to identify data categories and overarching themes.

**Results:**

Five major themes comprising multiple categories and codes emerged. These themes focused on the acceptability of the intervention, acceptability of the study, the perceived active ingredients of the intervention, the suitability of the intervention to adolescents’ lives, and recommendations for intervention improvement.

**Conclusions:**

Overall, Pain Squad+ and the pilot study protocol were acceptable to adolescents with cancer. Suggestions for intervention and study improvements will be incorporated into the design of a future randomized clinical trial (RCT) aimed at assessing the effectiveness of Pain Squad+ on adolescents with cancer health outcomes.

## Introduction

### Background

Up to 96% of adolescents with cancer experience pain related to the disease or associated invasive procedures and treatment [1]. Pain negatively impacts adolescent health-related quality of life (HRQoL) [2], impedes cancer recovery [3], interferes with activities of daily living [4], and results in long-term morbidity [5,6]. Pain may also represent a significant cost burden to the health care system and families [7], with pain being the most common reason for adults with cancer to utilize emergency health services [8-11]. Treatment advancements and health care system transformations have resulted in adolescents with cancer receiving much of their care on an outpatient basis [12]. This change may mean that a significant proportion of adolescent cancer symptoms (including pain) are experienced in an environment where management options may be suboptimally applied (eg, at home). In addition, adolescents with cancer have emerged as a childhood cancer subgroup with unique developmental characteristics and a unique cancer epidemiology [13-15]. These characteristics suggest that the cancer pain experience and appropriate management techniques may be different for adolescents when compared with younger children and adults [15-17].

In response to these issues, our team has developed a smartphone-based app, called Pain Squad+, that is capable of providing adolescents with real-time pain management support (Multimedia Appendix 1) [18,19]. The app integrates automated valid and reliable pain assessments [1] and personalized pain self-management advice with centralized decision support via a pediatric oncology-trained registered nurse to enable adolescents to better manage their pain in real time, regardless of their location (eg, hospital, home, school).

The development of Pain Squad+ has followed the phased approach to designing and evaluating complex interventions outlined by the UK Medical Research Council (MRC) [20]. In particular, we established a pain management evidence base for the intervention using a systematic review and expert consensus conference [21,22]. We then developed a Pain Squad+ software prototype and adapted the software to the needs of adolescents with cancer using iterative cycles of usability testing [18]. Next, the feasibility of implementing Pain Squad+ for a future randomized controlled trial (RCT) and preliminary intervention effect estimates were determined using a multicenter pre-post test pilot study [19]. The pilot study demonstrated a 77% (40/52) accrual rate, a 5% (2/40) withdrawal rate, technical issues to be experienced by 15% (6/40) of participants, 69 ± 38% adherence to pain reporting, and that Pain Squad+ was highly acceptable to adolescents with cancer, based on the quantitative Acceptability E-Scale (AES) [23]. Small to moderate effect sizes showed the potential impact of Pain Squad+ on key clinical outcomes, including pain intensity, HRQoL, and pain interference.

### Objective

The pilot study used a nested design with a qualitative exit interview conducted with adolescent participants once they completed or withdrew from the study [24]. The nesting of these qualitative interviews within the pilot was informed by the UK MRC framework and was intended as an in-depth process evaluation assessing our capacity to implement the intervention and clarifying mechanisms by which the intervention may work [20]. Our specific study aim was to elucidate the perceptions of adolescents with cancer who participated in these exit interviews as they related to Pain Squad+ acceptability, perceived helpfulness of Pain Squad+, and suggestions for app improvement, as well as satisfaction with the pilot study protocol. The ultimate goal of this research was to use these perceptions to inform changes to the Pain Squad+ app and study protocol before conducting an adequately powered RCT.

## Methods

Reporting in this paper is in accordance with the Consolidated Criteria for Reporting Qualitative Research (COREQ) tool [25] (Multimedia Appendix 2).

### Study Design

Guided by phenomenological inquiry [26], this semistructured interview-based qualitative study was nested within a pilot study. The pilot used a one-group pre-post study design examining the feasibility and preliminarily effectiveness of Pain Squad+ (ClinicalTrials.gov NCT02901834). The methods and results of the pilot study have been previously published [19].

### Setting and Participants

Forty participants were recruited for the pilot from two hematology/oncology divisions at Canadian pediatric tertiary care centers. Adolescents who met the following inclusion criteria were eligible: 12-18 years old, English-speaking and reading, undergoing cancer treatment, at least 2 months from diagnosis, and having self-reported pain of >3/10 at least once in the week before recruitment. Exclusion criteria were as follows: major comorbid conditions or receiving end-of-life care. Adolescents were primarily being treated on an outpatient basis, although some participants were hospitalized for periods during the study for disease, treatment, or complication-related reasons. We did not record the amount of hospitalized time. Enrollment in the pilot study was the only eligibility criterion for participation in qualitative interviews. To include the perspectives of adolescents who could have potentially varied in terms of their satisfaction with and commitment to the study, adolescents who withdrew from the pilot were eligible for participation in this study. A purposive maximum variation sampling strategy was used to recruit adolescents for the interviews. Specifically, adolescents who varied in terms of age, sex, diagnosis, and pilot-related outcomes (ie, quantitatively rated app acceptability, adherence, and pre- to poststudy change scores on health-related study outcome measures) were selected to participate. The number of adolescents interviewed was determined by the number of interviews needed to reach data saturation [27].

### Intervention

The Pain Squad+ app is an mHealth technology aimed at supporting the management of pain by adolescents with cancer and clinicians. The study team loaned adolescents participating in the pilot an Apple iPhone 6 loaded with the app. Using the app, adolescents completed a 22-item valid and reliable pain assessment [1] twice daily in the morning and evening for 28 days at times they specified. Three audible notifications occurring within a 30-min window signaled adolescents to complete each assessment and if a pain assessment was not completed within this time frame, it was considered missed. Adolescents were also able to complete an 8-item short-form pain assessment anytime between the morning and evening reports if pain was experienced. If an adolescent reported pain on any assessment, they received real-time pain self-management advice from the app according to an evidence-based investigator-developed algorithm. Recommendations could be pharmacological (eg, reminders to adhere to prescribed medications), psychological (eg, distraction techniques), or physical (eg, yoga instruction). In all cases, if pain advice was given, adolescents received a notification to complete another pain assessment in 1 hour and were provided with additional advice as appropriate. In addition to self-management advice from the app, email alerts related to clinically significant pain [28] (ie, 3 consecutive pain reports of ≥3/10) were sent to a study pediatric oncology-trained registered nurse. The nurse then contacted the adolescent and their medical team to discuss the case and initiate provider-driven intervention such as medication changes. To encourage adherence to pain assessment reporting and to using management advice, the Pain Squad+ app was gamified with adolescents playing the role of law-enforcement officers who receiving rewards for engagement with the app.

### Procedure

Ethics approval was obtained from the Research Ethics Boards at both study sites. Consent was obtained from interested and eligible adolescents. Parental consent for research involvement is not required in our jurisdiction. At enrollment, adolescents completed questionnaires on demographic information and comfort with smartphone devices. Disease-related data were collected from the adolescent’s health record. Adolescents also completed prestudy measures for the following preliminary effectiveness outcomes: (1) pain intensity (2) HRQoL, (3) pain interference, and (4) and pain management self-efficacy. Pain intensity was assessed using the Brief Pain Inventory, which assesses current pain and worst, least, and average pain in the preceding week on a 0 to 10 numerical rating scale [29,30]. Health-related quality of life was assessed using the Pediatric Quality of Life Inventory (PedsQL) 4.0, which is a valid and reliable 23-item instrument assessing general physical, emotional, social, and school function [31]. Higher scores represent better HRQoL. Pain interference was assessed using the Patient-Reported Outcomes Measurement Information System Pediatric Pain Interference Short-Form scale (PROMIS PPI-SF), which is a valid 8-item scale assessing the impact of pain on function. Participants scores are standardized and interpreted such that 60 represents high pain interference, 70 represents very high pain interference, 40 represents low pain interference, and 30 represents very low pain interference [4,32]. Pain management self-efficacy was assessed using an investigator-developed question as there were no appropriate psychometrically sound scales to assess this construct at the time of the study.

Adolescents were then asked to use the Pain Squad+ app for the 28-day period. Adherence to Pain Squad+ was defined as 100% when 56 reports (2 reports per day for 28 days) were completed. On day 29, each adolescent who did not formally withdraw participation was asked to complete poststudy outcome measures and the AES. The AES collected data on the degree to which Pain Squad+ was satisfactory to participants in terms of how difficult, helpful, enjoyable, and understandable it was to use, and how tolerable the amount of time required to complete it was. The possible total score range for the AES is 6 to 30, and greater scores indicate higher acceptability.

A semistructured, telephone-based, and audio-recorded interview was conducted with a sample of pilot study adolescents. These adolescents had completed the pilot between 1 day to 2 weeks before the interview. This time frame was selected to help ensure that adolescents could accurately recall the use of the app and participation in the study, while also being flexible to adolescents’ individual schedules. An interview guide based on a version that was previously and successfully used with adolescents with cancer [33] guided interviews, and field notes were taken. All interviews were conducted by one investigator (LJ) who is a pediatric oncology nurse and was familiar with many of the interviewed adolescents before the study. The interviewer was not blinded to participant characteristics or Pain Squad+ outcomes but used a bracketing procedure during interviews and analyses to minimize the impact of subjectivity on findings [34]. No other individuals besides the interviewer and adolescent were present for interviews. Interview topics included app acceptability, app ease of use and understanding, recommendations for app changes, the perceived clinical value of the app, and the acceptability of study involvement. All interviews were audio-recorded.

### Data Analysis

A trained transcriptionist transcribed interview audio-recordings verbatim. Data analyses began once the first interview was transcribed, allowing issues identified in early interviews to inform later interviews using constant comparative analyses [35]. The transcribed data were managed using NVivo 10.0 software (QRS International, Australia). Field notes were also included in the analysis. All data were read several times by 2 authors (LJ and VH) to obtain an overall understanding and identify data codes. The authors then independently coded the data using a line-by-line technique based on the study objectives. Using content analysis, codes were grouped into categories based on between-code relationships. Categories were generated until all data were classified under the existing categories [26,36,37]. Categories were then grouped into themes. Quantitative data were integrated into the analysis process to illustrate or clarify qualitative results using a mixed methods matrix approach [38]. It was intended that discrepancies in opinion regarding categories and themes would be resolved using group discussion with a third party; however, no discrepancies occurred. An audit trail consisting of analytical decisions was kept as a means to enhance validity [34].

## Results

### Participants and Interview Process

All 20 adolescents who were approached to participate in poststudy interviews agreed to do so. Nineteen out of 20 (95%) of these interviewed adolescents had completed the entire pilot study, and 1 out of 20 adolescents (5%) withdrew from participation after completing 5 days of the intervention due to worry about damaging the loaned phone. Multimedia Appendix 3 presents adolescent characteristics, Pain Squad+ adherence, AES scores, and health outcome change scores (from pre- to poststudy). Interview length ranged from 7 to 20 min, and no adolescents had any issues recalling the use of the app or study participation. Interview data were categorized into 5 main themes: acceptability of the intervention, potential active ingredients of the intervention, suitability of the intervention, recommendations for improvement, and acceptability of the study. Each theme comprised several categories and codes as shown in Table 1.

### Acceptability of the Intervention

#### General Impressions

All adolescents enjoyed using Pain Squad+. Adolescents stated the app was “fun” (female, 12 years), “really neat” (female, 15 years), and engaging.

One of the adolescents stated:

It’s really appealing to the eye. The color, the theme is good. The font. And it’s not really that hard to understand. The vocabulary is really straightforward and all of the things on it, like you know, the multiple-choice questions and the [visual analogue scale] sliders are really easy to use. And for cons? Don’t really think I can really think of any.

Male, 16 years

#### Usability

Every adolescent endorsed the ease of use of Pain Squad+:

It was really easy. It was very straightforward. It wasn’t really complicated. It was just like simplified so it was easy to use for little kids.

Male, 16 years

One adolescent specifically reported that because of her familiarity with smartphone apps, use of Pain Squad+ was not problematic:

I’m used to that stuff so it made it easy.

Female, 16 years

Thirteen participants discussed ease of understanding related to the pain assessment questions and pain management advice. For instance, one adolescent stated:

It was really straightforward. I think all the questions were worded well so you could like understand what they were getting at.

Female, 16 years

Two participants endorsed the app as efficient to complete:

It was good because it was really fast and easy.

Male, 14 years

#### Specifically Endorsed App Elements

Specific elements of Pain Squad+ that were well-liked or helpful to adolescents were the pain management advice, the design and gamification mechanics (endorsed by all participants), and the ability to use the app in real time and in any environment, especially when at home. Speaking about the pain self-management advice, one participant stated:

I thought the pain help ideas were really awesome. When they suggested like different things that you could do? Those were really helpful. And they had some [pieces of pain management advice] where they would suggest like relaxation and breathing, and how do I do that. And then you click on it and there is someone talking to you, walking you through it. Like how to relax. So that’s helpful because someone can tell you to relax, but you can just be sitting there like, “I don’t know how.”

Female, 16 years

Referring to the ability of the app to provide pain management advice at any time and in any environment, adolescents said:

I think overall it was good. Probably one of my favorite parts of it was that you could do the 8 questions in the middle (short-form pain assessment). That helps a lot.

Female, 12 years

There was stuff you could try at home and like do yourself. So I liked that.

Female, 15 years

#### Challenges

Sixteen adolescents discussed challenges they experienced with the app. Twelve participants stated that the 22-item morning and evening pain assessments included too many questions

It was okay and too much. Because sometimes people don’t want to like keep...um...doing the same thing over again...22 questions every time.

Male, 16 years

**Table 1:** Post-pilot study interview themes, categories, and codes.

Theme and category		Codes
**Acceptability of the intervention**		
	General impressions	Enjoyed use
	Usability	Easy to use Easy to understand Quick to complete
	Specifically endorsed app elements	Pain management advice Design and gamification mechanics Real-time any-environment reporting
	Challenges	Number of assessment questions Notifications Other technical problems
**Potential active ingredients of the intervention**		
	Self-management support	Engagement in self-management Self-monitoring of pain Patient-provider communication
	Study nurse support	Value of nurse Timing of nurse support
**Suitability of the intervention**		
	Impact on daily activities	Perceived burden
	Recommended usage	Appropriateness when ill Appropriateness when symptom-free Recommended usage
Recommended improvements		Additional self-management advice Additional gamified mechanics Tutorials on use Audio-visual assets Review data
**Acceptability of the study**		
	General impressions and specific likes	General impression Specific likes
	Motivation for participation	Altruism Novel experience Gamification
	Challenge	Study phone

Nine adolescents described challenges related to the frequency of pain assessment notifications. The notification issue appeared to be related to a technical problem with the app software server, where repeated notifications to complete the same pain assessment questionnaire were sent to some study phones, which was “annoying” (Female, 17 years) for adolescents:

Even if I did do my case (pain assessment), it would still just keep on giving notifications. And I know that after you say, “yes” to the case, (the app will) follow-up (on the severity of pain one hour later). But even if I would do the follow-ups, it would just keep on giving more and more (notifications).

Female, 15 years

Technical problems were the final app-related challenge cited. One adolescent described an issue likely related to the app being unable to connect to the internet:

Sometimes it was hard for me to do it. Like 2 or 3 times I couldn’t (use the app), because like as soon as I clicked on the app, the screen would just go white.

Male, 14 years

Two adolescents reported an issue related to not receiving scheduled reminders to complete pain assessments:

I think it was a problem with my version [of the app] but I wasn’t getting any reminders (to complete pain assessments)...So I set up just a regular alarm on the clock the phone has itself.

Male, 17 years

### Potential Active Ingredients of the Intervention

#### Self-Management Support

All participants reported that the ability of Pain Squad+ to support pain self-management was of therapeutic benefit. For example, one participant stated:

Yeah because being like an out-patient, you’re not at the hospital all the time. And you don’t want to call the doctor every time you have something as simple as a stomach ache when you know you got tips from the app to help.

Female, 14 years

The ability for adolescents to self-monitor their pain through routine assessments was also considered a valuable self-management feature of the app:

Yeah especially because it really helps me to track my pain and remember everything.

Male, 14 years

Improved awareness of pain was also a perceived benefit of the app:

Some of the questions were things that I didn’t really consider when I was thinking about my pain. So (Pain Squad+) helped with (recognizing how pain) affected me and all that.

Female, 16 years

Pain Squad+ helped to facilitate adolescents’ communication about pain and pain treatments with their medical team. Highlighting this finding, one adolescent said:

I think when I had a problem at home, like experiencing some kind of pain, after inputting it into the app and coming back to the hospital, talking to my doctor was easier.

Male, 17 years

#### Study Nurse Support

Ten participants discussed interaction with the Pain Squad+ study nurse as being of therapeutic benefit. Most adolescents appreciated knowing that the nurse was a component of the intervention even if they did not require pain management support from the nurse during the study. In particular, these adolescents viewed nurse involvement in Pain Squad+ as an extra layer of pain treatment support in the event that self-management strategies were ineffective:

I liked that when you answer questions if you go higher (in pain intensity rating), a nurse actually calls you and like asks about your pain. It’s actually a good thing because like if you actually have pain and you don’t know what to do, she can help you.

Male, 15 years

Three adolescents, however, reported not believing the nurse was essential to the Pain Squad+ intervention and that the recommended self-management strategies were sufficient to support pain treatment:

I had it pretty controlled on my own.

Female, 16 years

One adolescent commented that he experienced some difficulty in connecting with the nurse when she attempted to contact him:

The part where you get the advice from the nurse was good but then sometimes I would just miss her if I was out or my phone was on silent. So, it might be better if she left you a (text) message so that you could check what she was telling you to do.

Male, 14 years

### Suitability of the Intervention

#### Impact on Daily Activities

Seven participants commented that use of the app fits well with their usual daily activities, for example:

Oh it was good, it was good. It didn’t really take that much time and effort.

Female, 17 years

#### Recommended Usage

Nine participants stated that completing morning and evening pain assessments when not experiencing pain was not appropriate. In particular, one adolescent said:

Yeah, I didn’t really have pain all of the time. So, I think [the number of notifications] just wasn’t for me, but I’m sure for someone who’s going through the pain it’s going to be really helpful.

Male, 16 years

When asked about his lack of interaction with Pain Squad+, another adolescent said:

Like it got a little tiring sometimes. Because especially there are days I didn’t feel any problems, like have any problems, and I felt less inclined to actually finish the surveys.

Male, 17 years

### Recommendations for Improvement

Six adolescents suggested that the app include additional pieces of self-management advice. For example:

I think if there were more ways to help you manage your pain added on, then it would be more helpful.

Male, 14 years

These adolescents did not, however, have specific recommendations regarding which pieces of management advice should be added. Four participants recommended enhancements to the gamification mechanics of the app. One adolescent stated:

Um, maybe like something a little more than just like how you “level up” (by completing pain assessments and management advice). Just...more to do with that. A little more interactive kind of thing...like even more, fun.

Female, 14 years

Three participants recommended adding tutorials to Pain Squad+ on how to use the app. Another two adolescents recommended converting text-based content into an audio- or video-recorded format. For example:

Umm I think probably like uh a few videos from actual professionals, healthcare professionals, like doctors. Or like maybe like, err therapists, like massage therapists. If there was like maybe, for example like a massage therapist showing someone how to relieve pain in a certain area.

Male, 14 years

Finally, three adolescents suggested incorporating a capacity to visually review previously logged pain reports in order to track pain treatment progress.

### Acceptability of the Study

#### General Impressions and Specific Likes

Overall, adolescents enjoyed participating in the pilot, stating that the study was “really good” (Male, 15 years) and they “didn’t mind” (Female, 14 years) participating. Specifically, adolescents endorsed the ease of study participation and the ability to use the intervention for a time:

And I don’t know, I really liked...I just liked the app. I liked being able to record my pain, like without just writing it down in like a journal or something you know? I really liked how they gave me suggestions for like what I could do. I just liked it all, overall.

Female, 16 years

Two adolescents specifically cited completion of the outcome measures as a well-liked study component, with one participant stating:

I liked the questionnaires at the beginning and the end. Just to like compare sort of how you’re doing before and after.

Female, 15 years

#### Motivation for Participation

Two participants discussed the chance to potentially help other adolescents with cancer as their rationale for study involvement. For example:

I just liked, you know, contributing to the development of this app. It will be a huge help to other little kids going through cancer.

Female, 16 years

Another adolescent mentioned the novel experience of being a research participant as her motivation for participation. Finally, 6 participants stated that they were motivated to continue participation because of the gamification mechanics, for instance:

Yeah but it was pretty cool. It made you sort of want to do it more. “Okay I’ve got to do this because I want to get the next level.”

Female, 16 years

#### Challenge

The use of the study phone loaned to participants was a challenge for 5 adolescents. These participants already owned a smartphone and considered care and use of a second phone to be a burden:

Half the time, I wouldn’t even hear it because it would be in a different room or something and I just totally forget about it. If (Pain Squad+) was on my actual cell phone I probably would have done it more. But it wasn’t on my actual phone that I have on me all the time.

Female, 14 years

## Discussion

### Principal Findings

Pain is a problematic issue for adolescents with cancer [1,2] that may be mitigated with the rapid self-management support made possible by mHealth interventions [19]. Adolescents with cancer who participated in a pilot of the Pain Squad+ smartphone-based real-time pain self-management app reported that they generally liked the app and considered it helpful. In particular, adolescents reported that the app was easy to use and understand, supported the self-care of pain, and simplified patient-provider communication. The suitability of the app to adolescents’ lives and the acceptability of participating in the pilot were also shown. Finally, challenges related to app use and pilot participation that will inform intervention and study protocol changes were described.

Adolescents with cancer considered the intervention satisfactory, despite variations in age, sex, diagnosis, level of interaction with the app, and change scores on study outcome measures. As indicated by high levels of engagement with the app [19], adolescents liked the capacity of the app to support pain treatment *via* the advice provided (especially when they were outside the hospital), pain self-monitoring, and facilitation of communication with health care providers. These findings suggest that mHealth-based self-management of cancer symptoms in real time is amenable to young patients, echoing results from other mHealth-based disease self-management research conducted with youth [39-43]. Additionally, in accordance with previous research [33,44], all interviewed adolescents found the design and gamification mechanics attractive and acceptable. Gamification mechanics are a relatively novel addition to mHealth interventions, and there is still limited literature on benefits with respect to patient behavior change. However, the role these mechanics may play in incentivizing self-management behaviors in chronic conditions [45-47], combined with their acceptability to patients, makes the evaluation of their application to mHealth interventions an area for future exploration.

Challenges adolescents experienced with Pain Squad+ suggested that real-time symptom assessments questionnaires should be brief and notifications directing patients to interact with apps should be minimized. These findings are in contrast with previous studies conducted by our group that showed adolescents to report high acceptability with a 22-item pain questionnaire and the same notification schedule employed presently [1,33]. However, adolescents in the previous studies completed pain assessments only twice-daily for 14 days rather than for 28 days. Acceptability may have been adversely impacted by the increased length of pain reporting used in this study, as well as the technical issues we experienced related to the server sending some adolescents numerous notifications. Additionally, a number of adolescents suggested that it would be preferable to interact with the app only when symptomatic and that the scheduled morning and evening pain reports were not necessary. Creating an optimal balance between improving user engagement with symptom reporting and limiting alert fatigue [48] should be considered in developing real-time mHealth interventions. The technical problems experienced by adolescents also represented a challenge to intervention use. mHealth technical malfunctioning limits intervention usability [49] and also may represent a safety issue for users if the device does not collect, interpret, and deliver medical information to patients and health care providers as intended [50]. Pain Squad+ software modifications to ensure sound functioning and improve the user experience will be made before a subsequent Pain Squad+ RCT research is conducted.

Generally, adolescents suggested that both pain self-management advice and study nurse involvement contributed to the therapeutic benefit of the app. Previous qualitative research with adult cancer patients engaged in studies of similar real-time symptom management interventions also showed positive feedback related to the role of a health care provider in remotely monitoring symptoms [51,52]. Acceptability of the nurse suggests the role may be a valuable component of real-time symptom management interventions for cancer patients. During the pilot trial, however, only 38% (15/40) of participants actually interacted with the nurse [19], who represents a cost addition to the intervention. A planned Pain Squad+ RCT will, therefore, use a 3-arm design (ie, Pain Squad+ with nurse interaction, Pain Squad+ without nurse interaction, and control) to elucidate the therapeutic benefit and cost implications of clinician involvement.

On the basis of thematic analyses of interview data, satisfaction with the pilot study protocol in general, was high. Engagement in the study was considered easy and the chance to use the intervention was liked. Participant reports of altruism as the motivation for study participation agree with previous studies of adolescents with cancer engaged in research [53,54]. Other motivations for participation included the novelty of being involved in research and the gamification of the app. The cited challenge associated with study participation related to the loaned study phone. Fear of damaging the phone was the reason for the single episode of study attrition, and using the study phone if an adolescent owned their own phone was considered a burden. Given that 80% to 87% of Western adolescents currently own a smartphone [55,56], our future RCT will involve installing Pain Squad+ on adolescent’s personal devices and loaning phones only as necessary.

### Limitations

A potential limitation of this study involved the introduction of potential cognitive biases into study findings. In particular, experimenter bias may have been introduced as the study member who conducted the interviews and data analyses was a PhD student whose dissertation focused on the development of Pain Squad+. To minimize the effect of a bias whereby the student could be overly positive when interpreting participants’ remarks about the app and study, an additional study member independently conducted coding and analysis, and an audit trail detailing analyses decisions was kept. Bracketing or mentally suspending biases also minimized the impact of researcher subjectivity during data collection and analysis [34]. A second limitation relates to social desirability response bias whereby to provide a socially desirable response to the interviewer, adolescents could have denied negative thoughts and feelings about the app [57]. To minimize this effect, at the onset of interviews, adolescents were told that both positive and negative feedback was equally important. A final limitation relates to the pilot study design. Because we used a pre-post study pilot, we could not explore the experience of adolescents being randomized to treatment arms or the acceptability of participating in the control condition.

### Conclusions

This study has demonstrated the acceptability and perceived helpfulness of the Pain Squad+ intervention and pilot study protocol to adolescents with cancer, albeit with a few caveats and suggestions for improvement. This study also shows that both self-management and clinician support are generally considered as helpful ingredients in mHealth real-time symptom management interventions for adolescents. However, because not all adolescents agreed that the nurse involvement in the intervention was necessary, future research will elucidate the value of clinician support in improving pain-related outcomes for adolescents. Using the UK MRC framework, this qualitative research provides evidence that will be used to refine the Pain Squad+ intervention and study protocol to design a successful RCT. Specifically, we will modify the Pain Squad+ software by truncating the pain assessments, minimizing the number of notifications received by adolescents, and addressing technical problems related to accessing the app through the internet. Satisfaction with the protocol was generally demonstrated, but modifications to improve acceptability by including the capacity to install adolescents’ personal phones will be added before an RCT. Study findings have applicability to other researchers engaged in the design and development of mHealth- and internet-based interventions for youth with chronic and life-limiting health conditions.

## Appendix

Multimedia Appendix 1 Pain Squad+ pain management app screenshots. Top to bottom, left to right: (a) pain management screen; (b) sample of pain self-management recommendations; (c) detailed view of "Mental Games" pain management recommendation; (d) sample of "gamification" reward for adherence (ie, advancement through law-enforcement ranks); and (e) view of user placement within law-enforcement ranks to encourage adherence.

Multimedia Appendix 2 Consolidated Criteria for Reporting Qualitative Research (COREQ) checklist.

Multimedia Appendix 3 Characteristics of interviewed Pain Squad and participants.

## Abbreviations

AES: Acceptability E-Scale
HRQoL: health-related quality of life
UK MRC: United Kingdom Medical Research Council
RCT: randomized controlled trial
