# Supplementary figures and images for: Perceptions of Adolescents With Cancer Related to a Pain Management App and Its Evaluation: Qualitative Study Nested Within a Multicenter Pilot Feasibility Study
Source: JMIR Mhealth Uhealth. 2018 Apr 6;6(4):e80. doi: 10.2196/mhealth.9319 (PMC5910537; doi:10.2196/mhealth.9319)

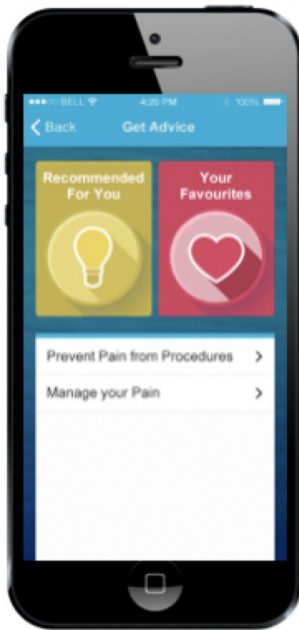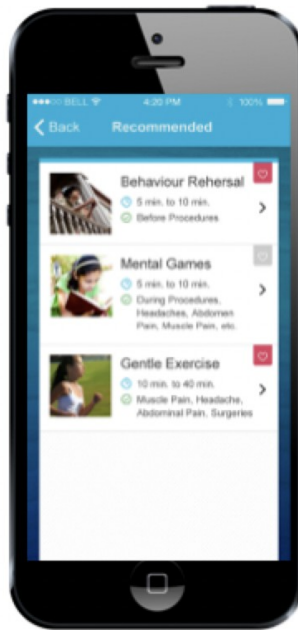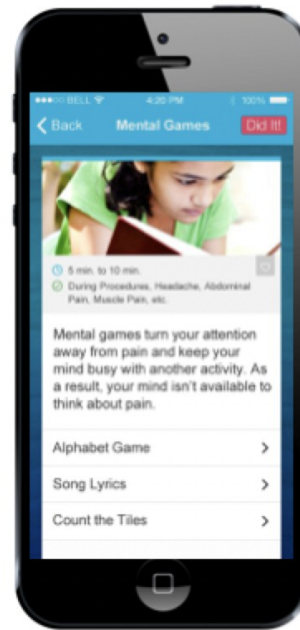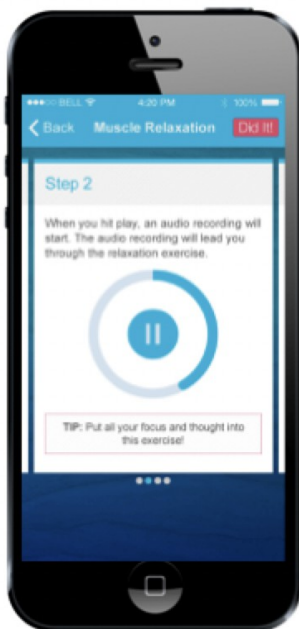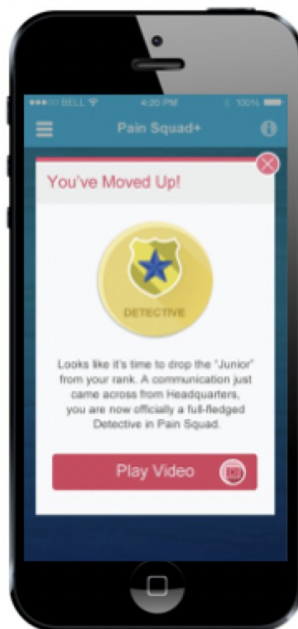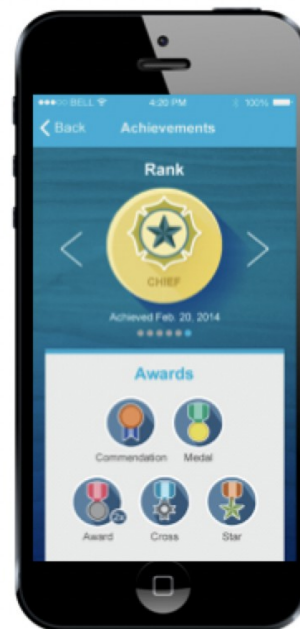

Supplement: Multimedia Appendix 1 [file mhealth_v6i4e80_app1.pdf]
