# Supplementary material for: Perceptions of Adolescents With Cancer Related to a Pain Management App and Its Evaluation: Qualitative Study Nested Within a Multicenter Pilot Feasibility Study
Source: JMIR Mhealth Uhealth. 2018 Apr 6;6(4):e80. doi: 10.2196/mhealth.9319 (PMC5910537; doi:10.2196/mhealth.9319)
Supplement: Multimedia Appendix 2 [file mhealth_v6i4e80_app2.pdf]

Item	Response
**Domain 1: Research team and reflexivity**	
Personal characteristics	
1. Interviewer /facilitator	The first author conducted all interviews
2. Credentials	Masters-level investigator from the field of nursing with previous experience in qualitative research
3. Occupation	The research team had representation from nursing, medicine, and engineering. Team member have expertise in pediatric oncology, pediatric pain, and mHealth development and testing.
4. Sex	The research team had both male and female representation.
5. Experience and training	The interviewer completed doctoral-level coursework in qualitative methodologies and methods and holds NVivo software certification. She had previously conducted numerous interviews with adolescents with cancer.
Relationship with participants	
6. Relationship established	The interviewer knew most of the adolescents prior to the interviews as she provided nursing care to them.
7. Participant knowledge of interviewer	All adolescents knew that this study was part of the interviewer's PhD research.
8. Interviewer characteristics	All adolescents knew that the interviewer's primary goal was to understand their impressions of the intervention and study in order to make improvements in preparation for a larger study.
**Domain 2: Study design**	
Theoretical framework	
9. Methodological orientation and theory	A content analysis approach was used.
Participant selection	
10. Sampling	A purposive maximum variation sampling strategy was used. Specifically, adolescents who varied in terms of age, sex, diagnosis, perceived Pain Squad+ acceptability and adherence, as well as baseline to post-study change scores on health-related study outcome measures were selected to participate.
11. Method of approach	The interviewer called adolescents who agreed to participate in interviewers on the telephone.
12. Sample size	20
13. Non-participation	Most participants from the pilot study (n=30; 91%) agreed in advance to participate in interviews. The interviewer contacted 20 of these adolescents and all participated in interviews.
Setting	

14. Setting of data collection	The interviewer was in her office and adolescents were either at home or in the hospital during the telephone interviews.
15. Presence of non-participants	The interviewer was alone during the interview. Adolescents could be in the presence of others during the interview, although were usually alone.
16. Description of sample	Adolescent characteristics are presented in Table 2.
Data collection	
17. Interview guide	An interview guide that was based on one previously used successfully with adolescents with cancer [33] was used. The guide was piloted for ease of understanding with 3 adolescents who met the inclusion criteria before formal testing. No modifications to the guide were required following piloting.
18. Repeat interviews	Repeat interviews were not conducted.
19. Audio-visual recording	Interviews were audio-recorded.
20. Field notes	Field notes were made after the interviews.
21. Duration	Interview length ranged from 7 to 20 minutes (M=11; SD=3).
22. Data saturation	Data saturation was considered to have been achieved when new interview data collected were redundant with those previously categorized (ie, after 20 interviews [27]
23. Transcripts returned	Transcripts were not returned to adolescents but were verified against audio-recordings for accuracy.
**Domain 3: Analysis and findings**	
Data analysis	
24. Number of data coders	Two study team members coded data (LJ and VH)
25. Description of coding tree	Initial codes were developed based on the study objectives, but codes were continually refined as simultaneous data collection and analyses were conducted. Table 3 shows the final set of data codes.
26. Derivation of themes	Codes were grouped into meaningful categories based on the relationships between codes. Categories continued to be generated until all study data were classified under the existing categories [26,37,38]. Categories were grouped into themes.
27. Software	The transcribed data were managed using NVivo 10.0 software [36] to code and annotate text.
28. Participant checking	Participants did not provide feedback on study results.
Reporting	
29. Quotations presented	Participant quotations are presented with an associated participant sex and age.
30. Data and findings consistent	The quotations presented exemplify the findings, which are consistent with the extant literature.
31. Clarity of major	Major themes are presented in Table 3 and body of the text.

themes	
32. Clarity of minor themes	Minor themes and diverse cases are presented in Table 3 and body of the text.
