# Supplementary material for: Perceptions of Adolescents With Cancer Related to a Pain Management App and Its Evaluation: Qualitative Study Nested Within a Multicenter Pilot Feasibility Study
Source: JMIR Mhealth Uhealth. 2018 Apr 6;6(4):e80. doi: 10.2196/mhealth.9319 (PMC5910537; doi:10.2196/mhealth.9319)
Supplement: Multimedia Appendix 3 [file mhealth_v6i4e80_app3.pdf]

Age (years)	Sex	Cancer diagnosis	AES score	*Adherence* (%)[a]	Outcome measure scores							
					BPI		PROMIS PPI-SF		PedsQL 4.0		Pain self-efficacy	
					Baseline	Poststudy	Baseline	Poststudy	Baseline	Poststudy	Baseline	Poststudy
15	F	ALL	22	28.5	4.0	3.8	64.4	65.4	52.2	53.3	2	1
12	M	ALL	23	83.9	5.8	2.8	63.4	63.4	59.7	67.4	missing	missing
16	F	Ovarian	23	108.9	5.5	6.5	65.4	67.6	36.8	41.5	missing	missing
13	M	LCH	missing	withdrew	missing	missing	missing	missing	missing	missing	missing	missing
14	M	ALL	24	58.9	7.8	3.3	75.0	61.4	26.0	43.5	3	3
14	M	Colon	27	37.5	0.8	0	65.4	34.0	33.7	75.0	missing	5
16	M	Lymphoma	29	71.4		1		6.3		-12.0	3	5
14	F	Lymphoma	25	92.9	2.3	1.3	50.6	44.3	75.0	87.0		0
12	M	ALL	28	51.8	2.3	3.8	59.5	64.4	55.4	45.7	5	5
16	F	AML	25	155.3	3.8	2.2	66.5	64.4	44.4	31.9	3	3
14	M	ALL	24	83.9	3.3	4.8	68.8	62.4	27.2	34.8	3	3
15	M	Lung	23	180.4	3.3	0	50.6	40.6	71.0	97.2	5	5
14	F	ALL	21	42.9	2.5	0	62.4	34.0	34.7	61.1	5	5
14	M	Lymphoma	24	58.9	2.8	1.0	54.7	50.6	73.6	73.9	3	3
14	F	Ewing sarcoma	25	21.4	2.3	0.5	42.7	missing	75.0	79.2	5	5
13	F	Colon	28	135.7	3.8	4.3	60.4	62.4	42.4	44.6	5	5
17	F	Lymphoma	missing	58.9	2.8	missing	61.4	missing	52.8	missing	3	missing

16	M	Germ cell tumor	missing	16.6	4.5	2.8	34.0	40.6	82.6	81.5	3	3
12	F	ALL	26	60.7	3	2.5	53.7	73.2	62.0	59.8	2	3
17	M	Lymphoma	22	67.9	3.3	0	60.4	34.0	47.2	64.1	3	3
Scale range			*6–30*			*0–10*		*3 –83*		*0-100*		*1–5*

[a] Percent of requested reports completed. Could be greater than 100% as some participants continued to complete pain reports on the app after the study had completed. Notes. AES = Acceptability E-Scale. ALL = Acute lymphoblastic leukemia. AML = Acute myeloid leukemia. BPI = Brief Pain Inventory. LCH = Langerhans cell histiocytosis. PedsQL 4.0 = Pediatric Quality of Life Inventory 4.0. PROMIS PPI-SF = Patient Reported Outcomes Measurement Information System Pediatric Pain Interference Scale Short Form.
